# The Influence of Electronic Health Record Use on Physician Burnout: Cross-Sectional Survey

**DOI:** 10.2196/19274

**Published:** 2020-07-15

**Authors:** Tania Tajirian, Vicky Stergiopoulos, Gillian Strudwick, Lydia Sequeira, Marcos Sanches, Jessica Kemp, Karishini Ramamoorthi, Timothy Zhang, Damian Jankowicz

**Affiliations:** 1 Centre for Addiction and Mental Health Toronto, ON Canada; 2 University of Toronto Toronto, ON Canada; 3 Krembil Centre for Neuroinformatics Centre for Addiction and Mental Health Toronto, ON Canada; 4 University of Waterloo Waterloo, ON Canada; 5 McMaster University Hamilton, ON Canada

**Keywords:** electronic health record, physician, burnout, psychiatry, medical informatics

## Abstract

**Background:**

Physician burnout has a direct impact on the delivery of high-quality health care, with health information technology tools such as electronic health records (EHRs) adding to the burden of practice inefficiencies.

**Objective:**

The aim of this study was to determine the extent of burnout among physicians and learners (residents and fellows); identify significant EHR-related contributors of physician burnout; and explore the differences between physicians and learners with regard to EHR-related factors such as time spent in EHR, documentation styles, proficiency, training, and perceived usefulness. In addition, the study aimed to address gaps in the EHR-related burnout research methodologies by determining physicians’ patterns of EHR use through usage logs.

**Methods:**

This study used a cross-sectional survey methodology and a review of administrative data for back-end log measures of survey respondents’ EHR use, which was conducted at a large Canadian academic mental health hospital. Chi-square and Fisher exact tests were used to examine the association of EHR-related factors with general physician burnout. The survey was sent out to 474 individuals between May and June 2019, including physicians (n=407), residents (n=53), and fellows (n=14), and we measured physician burnout and perceptions of EHR stressors (along with demographic and practice characteristics).

**Results:**

Our survey included 208 respondents, including physicians (n=176) and learners (n=32). The response rate was 43.2% for physicians (full-time: 156/208, 75.0%; part-time: 20/199, 10.1%), and 48% (32/67) for learners. A total of 25.6% (45/176) of practicing physicians and 19% (6/32) of learners reported having one or more symptoms of burnout, and 74.5% (155/208) of all respondents who reported burnout symptoms identified the EHR as a contributor. Lower satisfaction and higher frustration with the EHRs were significantly associated with perceptions of EHR contributing toward burnout. Physicians’ and learners’ experiences with the EHR, gathered through open-ended survey responses, identified challenges around the intuitiveness and usability of the technology as well as workflow issues. Metrics gathered from back-end usage logs demonstrated a 13.6-min overestimation in time spent on EHRs per patient and a 5.63-hour overestimation of after-hours EHR time, when compared with self-reported survey data.

**Conclusions:**

This study suggests that the use of EHRs is a perceived contributor to physician burnout. There should be a focus on combating physician burnout by reducing the unnecessary administrative burdens of EHRs through efficient implementation of systems and effective postimplementation strategies.

## Introduction

### Overview of Physician Burnout

Physician wellness is vital to the delivery of high-quality health care and greatly affects the performance of health care systems [1]. Organizations have started including wellness programs among their top priorities in an attempt to reduce physician burnout [2]—a work-related syndrome involving emotional exhaustion, depersonalization, and a sense of reduced personal accomplishment [3]. Physician burnout has been associated with career dissatisfaction [4], absenteeism and job turnover [5], reduced quality of care [6], and medical errors [7]. It is increasingly being measured, with high prevalence rates (78%) among American physicians [8], and almost 30% of Canadian physicians [9] have reported symptoms of burnout. The major contributors to physician burnout include *individual factors* (eg, age and education debt) [10] and *work factors* (eg, inefficient work processes, negative leadership, and limited interprofessional collaboration or advancement) [11]. Within psychiatry, workplace variables have been found to be a major stressor and may be more likely to perpetuate burnout [12]. Demands including navigating the working relationship with clients experiencing trauma—while often becoming the target of anger, hatred, and even violence—can be emotionally draining [13]. Moreover, burnout has also been known to span all phases of a physician’s career, including during medical school and residency, and a recent systematic review calculated a 33.7% burnout rate for psychiatry residents [14].

Among workplace inefficiencies, the use of health information technology such as electronic health records (EHRs) has been suggested to contribute to physician burnout in psychiatry [15,16]. With proper implementation, EHRs can improve the quality of health care by increasing time efficiency and guideline adherence and reducing medication errors [17]. However, the promise of improved quality of patient care through fast access to patient information and improved clinical decision-making support has not been attained in many health care organizations as the unintended consequences of EHRs proliferate [18].

These unintended consequences can be *technical* factors, such as poor software design, or *sociotechnical* factors, such as poor usability or workflow integration [19-22]. EHRs have often added to physicians’ cognitive load through excessive data entry requirements [23,24] and reduction of time spent with patients [25,26]. The provision of mental health care, in particular, poses an added level of complexity, including navigating multidisciplinary treatment plans, varying levels of care (ie, residential and partial hospitals) that do not fit neatly into the clinical or scheduling workflows [27], and the inability to capture and find important documentation [28,29]. Despite the challenges, the adoption of EHRs remains an important policy priority in most countries, showing a steady increase over the past 10 years and reaching an adoption rate of 75% in American [30] hospitals and 81% in Canadian [31] hospitals. Moving forward, the National Academy of Medicine calls for a *human-centered approach* to combat physician burnout by reducing unnecessary administrative burdens through improved design and implementation of technology and supportive regulatory policies [32].

Although there are numerous editorial and opinion pieces identifying EHRs as possible contributors to physician burnout [33,34], research in determining physicians’ perceptions of the impact of EHRs on burnout is scant [35]. In addition, there is a need to apply a variety of research methods to fully understand the complexity of the phenomenon to optimize technology and clinical workflows [35], given that previous studies used subjective, perception-based data for measuring both EHR-related stressors and burnout variables.

### Study Aims

This study aimed to (1) identify the extent of burnout and the perceived contribution of the EHR toward burnout among our population of physicians and learners (residents and fellows); (2) identify significant contributors of burnout and EHR-related burnout; (3) explore differences between physicians and learners among factors previously identified as contributing to EHR-related burnout (time spent, use, and documentation styles within the EHR; EHR proficiency and training; and perceived usefulness of EHR); and (4) compare self-reported perceptions on EHR usage metrics using log data.

## Methods

### Design, Setting, and Participants

This study used a cross-sectional survey design to gather the perceptions of physicians and learners and back-end EHR usage logs to gather use patterns. It was conducted at Canada’s largest academic mental health hospital located in Toronto, Ontario. At the time of the survey distribution, the Centre for Addiction and Mental Health had 407 physicians, 208 of which were considered full-time (ie, ones who had a *primary* appointment with the hospital). Among these physicians were hospitalists and psychiatrists spread across 7 different clinical divisions. In addition, the study included 53 residents and 14 clinical fellows. Of the 37,065 unique patients admitted in the hospital, 63.10% (23,388/37,065) were treated for an admission diagnosis falling within two groups: schizophrenia or/psychotic disorders and substance use disorders [36]. Physicians used a comprehensive EHR, which was implemented 5 years before this study.

Depending on the email address used for the electronic survey links, usage log data for 201 participants in May and 198 participants in June were identified, which were then used to compare metrics that were also asked within the survey.

Ethical approval was obtained from the organization’s quality improvement projects ethics review board.

### Data Collection

#### Survey

The survey was administered to the study’s target population of physicians and learners (residents and clinical fellows) between May 2019 and June 2019 by sending a link to the anonymous electronic survey via email. To maximize survey participation, weekly reminders were sent out for the duration of survey recruitment (6 weeks), and engagement methods, including advertising in physician newsletters, discussions at hospital-wide meetings, and resident lunches, were used.

Research Electronic Data Capture (REDCap)—a secure web app—was used to manage the survey [37]. The app collected information about demographics, practice setting and EHR-related usage, and burnout (Table 1) and open-ended responses to capture the respondents’ experience with the EHR and explore unique ways in which they use the EHR (Multimedia Appendix 1). The survey was developed using previous literature, including the study by Gardner et al [15] and KLAS Arch Collaborative Impact Report on Clinician Burnout [38], and was tested with physicians, researchers, and divisional chiefs, and their feedback was incorporated.

**Table 1:** Independent and dependent variables.

Variable type and category		Variables
**Independent**		
	Demographics	Age Gender (male, female, or nonbinary) Role (physician versus learners)
	Practice	Clinical academic division Length of practice Patient load (number of patients per week)
	EHR^a^-related factors	Time spent in EHR (time per patient and time after hours per week) Frustration and satisfaction with EHR Documentation styles (typing, back-end transcription, and voice recognition software) EHR proficiency EHR training needs Perceived usefulness of EHR (on improving communication, enabling high-quality care, and patient safety)
**Dependent**		
	Burnout	General burnout: Measured using a single question from the Mini-Z survey [39] (The Mini-Z is a 10-item instrument developed from the Physician Worklife Study [40]). This single question has been previously validated for physicians [41] against the detailed Maslach Burnout Inventory [3], and it achieved a sensitivity of 83.2% and specificity of 87.4% [41]. Respondents were asked to identify their symptoms of burnout based on a 5-point scale: (1) “I enjoy my work. I have no symptoms of burnout,” (2) “I am under stress, and don’t always have as much energy as I did, but I don’t feel burned out,” (3) “I am definitely burning out and have one or more symptoms of burnout, eg. emotional exhaustion,” (4) “The symptoms of burnout I am experiencing won’t go away. I think about work frustrations a lot,” and (5) “I feel completely burned out. I am at the point where I may need to seek help.” Participants considered as “burned out” include those having one or more symptoms of burnout (ie, a score of ≥3 on the above scale) Contribution of EHRs toward burnout: Measured by a single question where physicians and learners were asked “Do you think [EHR name] contributes to your symptoms of burnout?”, and responses were captured on a 4-point scale: “Always,” “Almost always”, “Some of the time,” and “Almost never.”

^a^EHR: electronic health record.

#### Usage Logs

As unique electronic survey links were used, a list of those who responded to the survey was assembled. Usage logs were extracted for all these individuals for the 2-month period (May-June 2019) of the survey administration. Although we were able to determine whether a participant had responded to the survey or not (a feature of REDCap), we were not able to identify their individual survey responses because of the anonymity of the survey. Therefore, variables extracted from usage log data were compared with responses from the survey in aggregate and not at an individual level. The data extracted were *EHR-related factors* including (1) number of patients seen per month, (2) time spent in EHR per patient, and (3) time spent in EHR after hours per month (details of the source of the back-end EHR analytics can be found in the study by Overhage et al [42]).

The inactive time when the physician was logged into the EHR but not actively engaged in using it (eg, typing) was excluded from the analyses, and *after-hours* were defined as 6 PM to 6 AM and weekends—similar to how it was defined in the survey.

### Data Analysis

#### Survey

Descriptive statistics were calculated for all numeric and categorical variables. The association of independent variables (including demographics, practice styles, and EHR factors) with variables measuring burnout was analyzed using Fisher exact tests and chi-square tests (when the content table was too large for the exact Fisher test to be calculated). Fisher exact tests were used to identify differences between physicians and learners for the following variables (counts of 5 or lower in chi-square tables): age, patient load, time spent in EHRs per patient, frustration with EHR, satisfaction with EHR, perceived usefulness (on improving communication within the circle of care, enabling delivery of high-quality care, and keeping patients safe), and documentation type. All descriptive and chi-square analyses were conducted using the Statistical Package for the Social Sciences software [43], and Fisher exact tests were conducted in R [44]. When significance at. 05 level was not achieved, no *P* values were reported within the study’s results.

For open-ended survey responses, inductive content analysis was used [45]. After dividing the responses into two groups (low and high satisfaction with EHR based on a quantitative rating scale), the data were read and coded to capture key thoughts or concepts. Investigator triangulation was used to refine the coding scheme, and one investigator proceeded to code all data. Emergent codes were clustered into broader subcategories or themes.

#### Comparison: Self-Reports and Usage Log Data

As back-end EHR usage log data were extracted on a monthly basis, the 2 survey variables (patients seen per week and time spent in EHR after hours per week) were changed from *weekly* to *monthly* to ensure practical comparison. Descriptive statistics gathered from the survey responses and usage logs of all respondents were compared.

## Results

### Participant Profile

Demographic and practice characteristics of the study population are shown in Table 2. The survey was answered by 176 physicians and 32 learners. Response rates were 43.2% for physicians (full-time: 75% and part-time: 10%) and 47.7% for learners (fellows: 86% and residents: 40%). A total of 44.3% (78/176) of physicians and 50% (16/32) of learners were female; 46.0% (81/176) of physicians were in the 0 to 10 years practice timeframe and 26.1% (46/176) practiced for 21 years or more. Physicians saw a mean of 27 patients (median 25) per week and learners saw a mean of 14 patients (median 15).

**Table 2:** Demographic and practice characteristics, by experience level.

Demographics		Total sample (N=208), n (%)	Physicians (n=176), n (%)	Learners (residents and fellows; n=32), n (%)
**Age (years)**				
	<30	17 (8.2)	4 (2.3)	13 (41)
	31-40	81 (38.9)	63 (35.8)	18 (56)
	41-50	59 (28.4)	58 (33.0)	1 (3)
	51-60	23 (11.1)	23 (13.1)	0 (0)
	≥61	28 (13.5)	28 (15.9)	0 (0)
**Gender**				
	Female	94 (45.2)	78 (44.3)	16 (50)
	Male	105 (50.5)	89 (50.6)	16 (50)
	Gender fluid or nonbinary or two-spirit	1 (0.5)	1 (0.6)	0 (0)
	I prefer not to answer	8 (3.8)	8 (4.5)	0 (0)
**Practice setting^a^**				
	Emergency mental health	40 (19.2)	22 (12.5)	18 (56)
	Inpatient mental health	82 (39.4)	69 (39.2)	13 (41)
	Outpatient mental health	159 (76.4)	131 (74.4)	28 (88)
	Telehealth	25 (12.0)	21 (11.9)	4 (13)
	Outreach	8 (3.8)	8 (4.5)	0 (0)
	Unknown	2 (1.0)	2 (1.1)	0 (0)
**Patient load/week**				
	≤10	53 (25.5)	39 (22.2)	14 (43)
	11-20	55 (26.4)	39 (22.2)	16 (50)
	21-30	46 (22.1)	45 (25.6)	1 (3)
	≥31	53 (25.5)	52 (29.5)	1 (3)
	Unknown	1 (0.5)	1 (0.6)	0 (0)

^a^Practice setting was a multi-select question.

### Burnout and the Perceived Contribution of the Electronic Health Record Toward Burnout

A total of 25.6% (45/176) of all physicians and 19% (6/32) of learners identified as having one or more symptoms of burnout, as measured using the single-item measure from the Mini-Z. When asked about EHR contributing to burnout, 69.3% (122/176) of physicians and 67% (22/32) of learners reported feeling that the EHR always or almost always contributes to their symptoms of burnout. Within the subset of those individuals who experienced one or more symptoms of burnout (n=51), this perception of the EHR contributing to burnout was slightly more prevalent (155/208, 74.5%; Table 3).

**Table 3:** Burnout prevalence, by experience level.

Burnout measure		Total sample (N=208), n (%)	Physicians (n=176), n (%)	Learner (residents and fellows; n=32), n (%)
**General physician burnout**				
	1: “I enjoy my work. I have no symptoms of burnout”	45 (21.6)	38 (21.6)	7 (22)
	2: “I am under stress, and don’t always have as much energy as I did, but I don’t feel burned out”	111 (53.4)	92 (52.3)	19 (59)
	3: “I am definitely burning out and have one or more symptoms of burnout, e.g. emotional exhaustion”	35 (16.8)	31 (17.6)	4 (13)
	4: “The symptoms of burnout I am experiencing won’t go away. I think about work frustrations a lot”	15 (7.2)	13 (7.4)	2 (6)
	5: “I feel completely burned out. I am at the point where I may need to seek help.”	1 (0.5)	1 (0.6)	0 (0)
	Unknown	1 (0.5)	1 (0.6)	0 (0)
One or more symptoms of burnout^a^		51 (24.5)	45 (25.6)	6 (19)
**EHR^b^ contributing to physician burnout**				
	Always/almost always	144 (69.2)	122 (69.3)	22 (67)
	Some of the time/almost never	63 (30.3)	53 (30.1)	10 (31)
	Unknown	1 (0.48)	1 (0.57)	0 (0)

^a^General physician burnout score ≥3.

^b^EHR: electronic health record.

### Significant Contributions to Burnout

**Figure 1:**
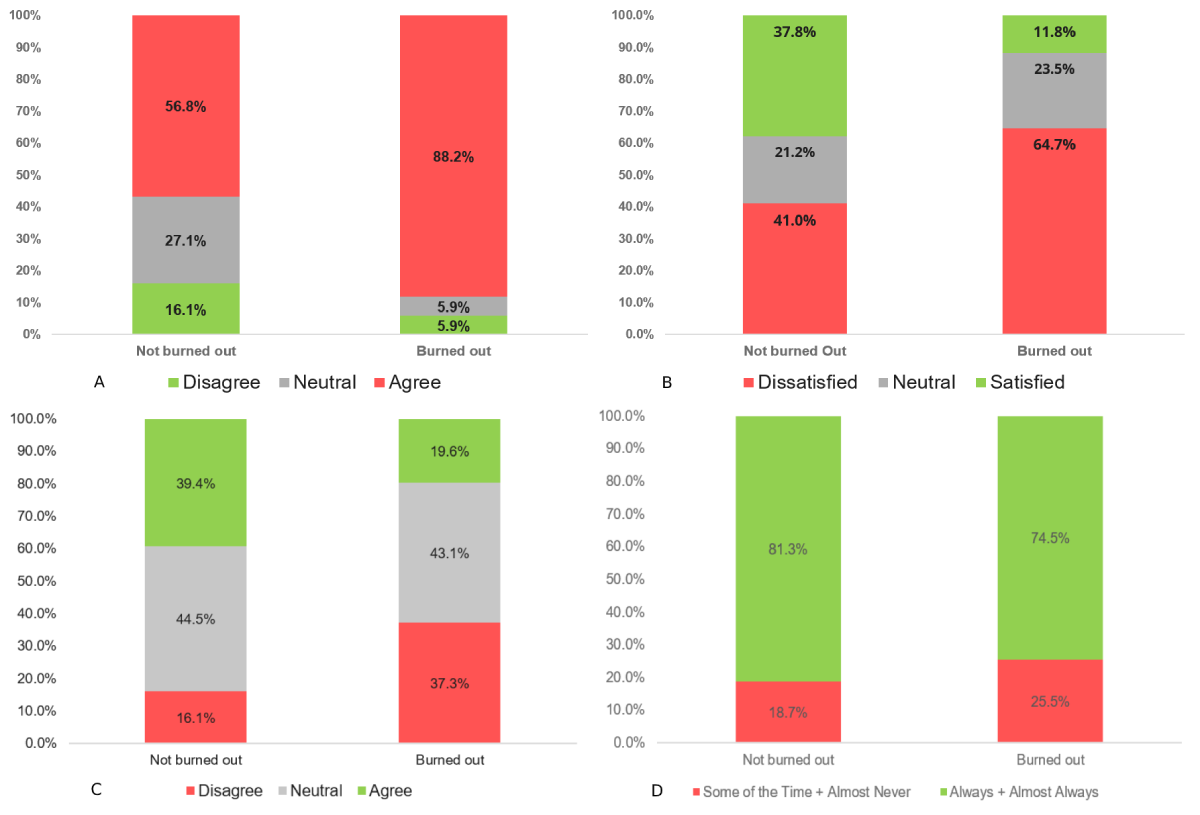
Significant contributors to physician burnout. A: [EHR name] adds to my daily frustration (*P*<.001); B: How would you rate your satisfaction with [EHR name]? (*P*<.001); C. [EHR name] helps keep my patients safe (*P*<.002); D: Do you feel communication regarding [EHR name] changes are efficient? (*P*<.047).

Physicians and learners with higher levels of frustration and lower satisfaction with the EHR were significantly more burned out (Figure 1). Other variables that were significantly associated with burnout included participants’ perceptions of the EHR on keeping their patients safe (*P*=.002) and whether physicians perceived communication regarding EHR upgrades as efficient (*P*=.047).

Variables that had a significant association with perceptions of EHR contributing to burnout were low satisfaction with the EHR (*P*<.001) and frustration with the EHR (*P*<.001; Figure 1). Similarly, as with burnout, those who were content with communication surrounding EHR updates were also significantly less likely to perceive the EHR as a contributory factor to burnout (*P*=.003). Finally, those who were more proficient with the EHR were also significantly less likely to perceive it as contributing to their burnout (*P*=.01).

The experiences of physicians and learners who had *low satisfaction* with the EHR focused on usability issues and unintended consequences of the EHR on patient care. A total of 39 individuals reported usability issues such as it being nonintuitive, having too many clicks, or not being user-friendly, with one participant referring to it as “death by a thousand clicks” (Participant #94). Moreover, 48 respondents discussed difficulties with finding or retrieving information, including inaccessible documentation. Respondents also discussed time sinks because of the system being “slow” and “clunky” and noted the impact that technology has on direct patient care, such as “...has a negative impact on...the amount of quality face-to-face time I can spend with patients” (Participant #118).

Respondents with *high EHR satisfaction* used workarounds to complete tasks in the EHR, such as “type long consult notes in [Microsoft] word then copy” (Participant #33) or “enter my appointments in my Outlook calendar...” (Participant #66). Others discussed their knowledge of customization, such as “know how to insert personal short cuts” (Participant #103), or the use of back-end dictation or “use [voice recognition software] exclusively instead of typing progress notes” (Participant #125). Satisfied participants thought that the EHR allows for “communication with other care providers” and has “the ability to forward things easily to care providers in the circle of care” (Participant #168).

### Physician and Learner Differences for Electronic Health Record–Related Factors

#### Time Spent, Use, and Documentation Styles Within the Electronic Health Record

Time spent, use, and documentation styles were not significantly different between those participants who were burned out and those who were not burned out. There were significant differences between physicians and learners with respect to the time spent on the EHR per patient, where 47% (15/32) of learners spent over 50 min per patient compared with only 16.5% (29/176) of physicians spending the same amount of time (*P*=.03). In total, 84.1% (148/176) of physicians and 78% (25/32) of learners reported spending time on the EHR after hours, with 22.2% (2/208) of respondents spending 4 or more hours per week.

Compared with physicians (96/176, 54.5%), a greater proportion of learners (24/32, 75%) document in the EHR through direct typing only. In contrast, 2.8% (5/176) of physicians rely solely on dictation through back-end transcription or voice recognition. None of the learners used dictation only to document in the EHR. Leaners that did not use direct typing as their documentation style (8/32, 25%) used a combination of direct typing and dictation, whereas 41.5% (73/176) of physicians used a combination of documentation styles.

#### Electronic Health Record Proficiency and Training

A total of 31.3% (55/176) of physicians and 41% (13/32) of learners felt that their initial EHR training prepared them well, and a large portion of physicians and learners (89/176, 50.6% and 13/32, 41%, respectively) felt they had ongoing training available to meet their needs. In addition, more than half of the physicians (94/176, 53.4%) and learners (22/32, 69%) felt proficient in their use of the EHR. The majority of physicians (14/176, 79.5%) and learners (24/32, 75) reported that communication regarding changes to the EHR was effective.

#### Perceived Usefulness of Electronic Health Records

A total of 62.5% (110/176) of physicians and 72% (23/32) of learners indicated that the EHR adds to their daily frustration (Table 4). Although 51.1% (90/176) of physicians felt that the EHR improved communication in their circle of care, only 34% (11/32) of learners agreed with this statement. With regard to the impact of the EHR on improving patient safety, 38.1% (67/176) and 40.3% (71/176) of physicians agreed with the statement or were neutral, respectively. In contrast, 63% (20/32) of learners felt neutral about this statement and 13% (4/32) agreed. Physicians had a significantly more positive perspective on the EHR in terms of quality of care (*P*=.007), with 38.6% (68/176) agreeing that it enabled them to deliver high-quality care, compared with only 9% (3/32) of learners.

**Table 4:** Electronic health record factors, by experience level.

Demographics		Total sample (N=208), n%	Physicians (n=176), n (%)	Learner (residents and fellows; n=32), n (%)
**Satisfaction with EHR^a^**				
	Very satisfied/somewhat satisfied	97 (46.6)	1 (43.2)	21 (66)
	Neither satisfied nor dissatisfied	45 (21.6)	37 (21.0)	8 (25)
	Somewhat dissatisfied/very dissatisfied	65 (31.3)	62 (35.2)	3 (9)
	Unknown	1 (0.5)	1 (0.6)	0 (0)
**Frustration with EHR**				
	Disagree	26 (12.5)	25 (14.2)	3 (9)
	Neutral	45 (21.6)	39 (22.2)	6 (19)
	Agree	133 (63.9)	110 (62.5)	23 (72)
	Unknown	2 (1.0)	2 (1.1)	0 (0)
**Time spent in EHR per patient (min)**				
	≤10	32 (15.4)	31 (17.6)	1 (3)
	11-20	56 (26.9)	51 (29.0)	5 (16)
	21-50	74 (35.6)	63 (35.8)	11 (34)
	≥50	44 (21.2)	29 (16.5)	15 (47)
	Unknown	2 (1.0)	2 (1.1)	0 (0)
**EHR improves quality of care**				
	Disagree	46 (22.1)	34 (19.3)	12 (38)
	Neutral	89 (42.8)	72 (40.9)	17 (53)
	Agree	71 (34.1)	68 (38.6)	3 (9)
	Unknown	2 (0.9)	2 (1.1)	0 (0)

^a^EHR: electronic health record.

### Self-Reported Perceptions and Electronic Health Record Usage Log Data Comparison

As gathered from usage logs, the median *number of patients seen per month* for all the survey respondents over the months of May and June 2019 was 60 patients (May, N=201, June, N=198), compared with the self-reported median of 80 patients (N=207). The median *time spent on EHRs per patient* for all survey respondents for the months of May and June 2019 was 16.4 min (May, N=201, June, N=198), compared with the self-reported median of 30 min (N=206). The median *time spent on the EHR after hours* (defined as 6 PM to 6 AM and weekends) for all survey respondents for the months of May and June 2019 was 2.37 hours (May, N=201, June, N=198), compared with the self-reported median of 8 hours (N=201).

## Discussion

### Principal Findings

Although overall satisfaction with EHRs remains low, reverting to paper documentation is not a viable alternative. This study adds to a growing body of evidence calling for a focus on EHR improvement [46]. The study data demonstrate that 67% of learners and 43% of physicians were satisfied with the system, which is comparable with other studies [47]. Among those who perceived the EHR in a negative light, a majority (>65%) of the respondents expressed dissatisfaction with the EHR (n=97).

### Burnout and the Perceived Contribution of the Electronic Health Record Toward Burnout

This study helps in understanding physician burnout attributed to technology within the Canadian mental health context. Although the general burnout rate of physicians and learners (24.6%) was comparable with the Canadian national average (30%) [9], our survey found that the majority (69.6%) of physicians and learners attributed EHR to their symptoms of burnout, even when they did not identify as being burned out. Although other institutions have completed surveys to examine the role of technology in physician burnout [15,16,48], this study adds to the existing literature, providing data from a different geography and a robust baseline at our facility. Measuring burnout rates, as well as the significant EHR-related contributors to burnout within the hospital, have direct implications on practice. Our organization has created a multipronged approach toward improving physicians’ experience with the EHR, which includes direct feedback channels, improved education and communication around EHR updates, implementing speech recognition technology, and developing physician efficiency dashboards. Having a strong baseline measure of burnout allows us to measure the short- and long-term impact of initiatives at our hospital which aim to reduce physician burnout.

#### Significant Contributors to Burnout

Gardner et al [15] found that those who spent excessive time on the EHR at home had a 1.9 times higher rate of burnout, and Privitera et al [48] found that EHR use at home increased burnout by 46% within their population. However, this study found no significant differences in the time spent after hours between those respondents who were and were not burned out.

Another previously affiliated factor with EHR satisfaction was gender, with men reporting significantly higher EHR workload stress than women [49]. This study did not find any significant differences between men, women, or nonbinary individuals when it came to satisfaction and frustration with EHR, as well as other EHR-related factors.

The results from this study did identify low proficiency with the EHR as a significant factor that leads physicians and learners to perceive the EHR as contributing toward their burnout, which supports the hypothesis that improved education and training can help in reducing this negative perception. Research by Dastagir et al [50] demonstrated the impact of proficiency training on significant improvements in self-reported efficiency and satisfaction, which could eventually have an effect on burnout.

#### Physician and Learner Differences for Electronic Health Record–Related Factors

In addition, this study found significant differences between physicians and learners with respect to the time spent in the EHR per patient, with a higher number of learners spending >50 min. Such a difference could be attributed to learners getting used to a new EHR system (as they often work with several EHRs across the various training sites) and mastering clinical practice and documentation standards. They could also be tasked with doing more designated EHR work, including documentation and orders, allocated by their supervising physician. Similar results demonstrating the extent of indirect patient care that residents take on were found in a time-motion study conducted by Penn Medicine and John Hopkins University. The study found that the residents spend almost 66% of their time interacting with patients’ medical records or documentation [51].

#### Self-Reported Perceptions and Electronic Health Record Usage Log Data Comparison

This study found that perceptions of time spent in the EHR after hours were much higher than the actual time spent, as gathered by back-end usage logs, with an >5-hour difference between these 2 averages. It is possible that the time spent after hours for logging in and out of the system, on email, and for other digital administrative activities could be included within participants’ perceived estimates. This difference resembles previous research that has found overestimations of 1.83 hours in learners and up to 4.04 hours in attending physicians [52]. This study’s respondents also overestimated the time spent in the EHR per patient compared with how long they spend according to back-end usage logs (14-min difference), which could be because of interruptions in the workflow. It is important to note that, in general, employees have been shown to overestimate the hours that they work [53].

This discrepancy between self-reported and back-end usage log data has implications for future research, where a combination of methods should be used for studying the link between EHR-related stressors and physician burnout. Although burnout is primarily measured through perceptions, the stressors related to the EHR, such as time spent in the EHR after hours, primary documentation method, and amount and frequency of training, can all be measured through more objective means.

### Limitations

Due to the nature of this study, we were only able to report on associations between variables rather than causal relationships.

To improve the understanding of the complexity of EHR use, we used back-end EHR usage logs. However, because of the anonymity of the survey, we were unable to compare self-reported data with usage logs on an individual basis, and we could only provide an aggregate comparison of 3 measures. Furthermore, usage logs can lack the vital context around clinical workflows, and there has been varied validity and sensitivity of using such logs for mapping out clinical activity [54]. Validation of back-end EHR usage logs through direct observation was not carried out within this study environment; however, this analytics system has been used in previously published literature describing physicians’ EHR usage [42].

Despite numerous discussions and publications, there are striking differences in the understanding of what constitutes burnout and substantial variability in prevalence estimates of burnout among physicians [55]. This study used a single question from the Mini-Z, which was previously validated by physicians, and yielded results similar to those of the more commonly used Maslach Burnout Inventory [3].

Finally, because of the heterogeneity of EHRs, implementation practices, training, and organizational contexts, there exists a potential limitation in generalizing such results to other contexts.

### Conclusions

This work is the first step in better understanding EHR-related physician burnout in a Canadian academic mental health environment, where we measured general burnout rates and its perceived link to EHR use through a survey that gathered self-perceptions. In addition, we compared self-perceptions with a back-end usage log for 3 important metrics and found that participants tended to overestimate their time spent on the EHR. This finding provides a valuable contribution toward the methodology for studying physician burnout and demonstrates the need to combine self-reported perceptions with objective data sources.

The contribution of this study to the literature on physician burnout demonstrates the importance of increasing end-user satisfaction and minimizing end-user frustration with the EHR, both significant factors that were associated with burnout within the study population. The results of this study emphasize the value of developing *human-centered* effective strategies to improve physicians’ experiences with EHRs, including efficient communication about EHR upgrades. Measuring burnout and understanding the impact of EHR-related stressors within the study population serves as a strong baseline, allowing us to measure the short- and long-term impact of multiple initiatives underway at our hospital aimed at reducing physician burnout.

## Appendix

Multimedia Appendix 1 Detailed survey questions.

## Abbreviations

EHR: electronic health record
REDCap: Research Electronic Data Capture
