# Supplementary material for: The Influence of Electronic Health Record Use on Physician Burnout: Cross-Sectional Survey
Source: J Med Internet Res. 2020 Jul 15;22(7):e19274. doi: 10.2196/19274 (PMC7392132; doi:10.2196/19274)
Supplement: Multimedia Appendix 1 [file jmir_v22i7e19274_app1.pdf]

Survey Questions

Section 1: Demographics

Q1. I am a:
○ Staff physician
○ Fellow
○ Resident

Q2. How would you describe your clinical activities?
☐ Inpatient Physician
☐ Outpatient Physician
☐ Emergency Department
☐ Telehealth
☐ Outreach
(Please select all that apply.)

Q3. What is your current academic job description?
○ Clinician Administrator
○ Clinician Educator
○ Clinician Investigator
○ Clinician Scientist
○ Clinician Teacher
○ Clinician in Quality & Innovation
○ None of the above

Q4. What is your primary division?
○ Addictions
○ Adult Neurodevelopment and Geriatric Psychiatry
○ Child and Adolescent Psychiatry
○ Forensics
○ General Adult and Health Systems Psychiatry
○ Medicine in Psychiatry
○ Schizophrenia

Q5. How long have you been practicing as a physician?
○ 0-5 years
○ 6-10 years
○ 11-15 years
○ 16-20 years
○ 21-25 years
○ 26+ years

Q6. How frequently do you provide clinical care at the hospital?
○ Part-time
○ Full-time

Q7. What is your age?
○ < 30
○ 31-40
○ 41-50
○ 51-60
○ 61+

Q8. Please select the option that best describes you:
○ Woman
○ Man
○ Gender-fluid, non-binary, and/or Two-spirit
○ I prefer not to answer

Section 2: Use of EHR

Q9. Estimated number of patients seen per week.

____

Q10. Estimated time spent in EHR per patient seen. This includes: documentation, orders, chart review and other uses of EHR. (Please provide a value in minutes.)

____

Q11. How much time do you spend per week using EHR after hours? (After hours defined as after 6 PM/before 6 AM or during the weekends, not including duty doctor or physician on call shifts) (Please provide a value in minutes or hours.)

____

Q12. Did you provide a value in minutes or hours for the previous question?
○ Minutes
○ Hours

	Strongly Disagree	Disagree	Neutral	Agree	Strongly Agree
Q13. EHR adds to my daily frustration.	○	○	○	○	○
Q14. EHR improves communication within the . circle of care.	○	○	○	○	○
Q15. EHR enables me to deliver high quality care.	○	○	○	○	○
Q16. EHR helps keep my patients safe.	○	○	○	○	○

Section 3: Training

	Strongly Disagree	Disagree	Neutral	Agree	Strongly Agree
Q17. I feel proficient with my the EHR use.	○	○	○	○	○
Q18. My initial training prepared me to use the EHR well.	○	○	○	○	○
Q19. Ongoing training for my needs is available.	○	○	○	○	○

Section 4: Documentation and Communication

Q20. What methods do you use to document?
☐ I type directly into EHR
☐ I use dictation/transcription services (back end dictation)
☐ I use Voice Recognition software (front end dictation)
(Please select all that apply.)

Q21. In your experience with Voice Recognition software, the tool...
☐ Has improved the quality of my clinical documentation
☐ Has decreased the time spent documenting client care
☐ Is acceptably accurate at transcribing my speech
☐ I would benefit from ongoing training
☐ I would benefit from increased accessibility/mobility of the tool
☐ None of the above
(Please select all that apply)

Q22. Do you feel communication regarding EHR changes are efficient?
○ Always
○ Almost always
○ Some of the time
○ Almost never

Section 5: Burnout

Q23. Identify your symptoms of burnout.
○ I enjoy my work.I have no symptoms of burnout.
○ I am under stress, and don't always have as much energy as I did, but I don't feel burned out.
○ I am definitely burning out and have one or more symptoms of burnout, e.g., emotional exhaustion.
○ The symptoms of burnout that I am experiencing won't go away. I think about work frustrations a lot.
○ I feel completely burned out. I am at the point where I may need to seek help.

Q24. Do you think EHR contributes to your symptoms of burnout?
○ Always
○ Almost always
○ Some of the time
○ Almost never

Section 6: General Feedback

Q25. How would you rate your satisfaction with EHR?
○ Very dissatisfied
○ Somewhat dissatisfied
○ Neither satisfied nor dissatisfied
○ Somewhat satisfied
○ Very satisfied

Q26. What do you believe you do differently from your peers while using the EHR?

____

Q27. Tell us about your experience with the EHR.

____

Q28. Do you have any other comments?

____
